# Supplementary material for: Two Old Wild-Type Strains of Drosophila melanogaster Can Serve as an Animal Model of Faster and Slower Aging Processes
Source: Insects. 2024 May 3;15(5):329. doi: 10.3390/insects15050329 (PMC11122303; doi:10.3390/insects15050329)
Supplement: Supplementary file 1 [file insects-15-00329-s001.zip › insects-2949179-supplementary.pdf]

Supplementary Material

to

“Two old wild-type strains of *Drosophila melanogaster* can serve as an animal model of faster and slower aging processes”

Supplementary Material 1: Tables S1-S5 and Figures S1-S10

Table S1. Survival time under 29°C.

(A) Survival time in parent strains, days

Sex	Dose	Harwich Mean	Harwich SEM	Harwich Median	Harwich SEM	Canton-S Mean	Canton-S SEM	Canton-S Median	Canton-S SEM	Long Rank, χ^2
Averaged	x0	22.1	0.5	22	0.6	28.2	0.6	31	0.2	106.4***
Male	x0	26.1	0.5	26	0.5	34.7	0.7	39	0.0	106.5***
	x1	22.0	0.6	19	0.9	33.4	1.0	39	0.0	118.6***
	x10	19.9	0.3	19	0.3	24.1	0.7	26	0.7	38.6***

(B) Survival time in parent strains and bidirectional crosses, days

Sex	F	Me	Harwich Me	Harwich SEM	Canton-S Me	Canton-S SEM	♀H♂C-S Me	♀H♂C-S SEM	♂C-S♀H Me	♂C-S♀H SEM	χ^2
Male	F1	Mean	24.0	0.6	29.0	0.7	33.2	0.3	32.8	0.5	349.7***
		Median	24	0.7	31	1.2	35	0.3	35	0.1	
	F70	Mean	20.2	0.7	27.4	0.9	25.3	0.8	28.1	0.7	94.2***
		Median	15	0.7	31	0.2	27	0.4	31	0.2	
Female	F1	Mean	21.2	0.3	28.8	0.8	34.7	0.4	32.9	0.6	290.3***
		Median	22	0.0	27	2.2	36	0.1	36	0.1	
	F70	Mean	27.4	0.7	33.5	0.8	34.7	1.0	33.5	0.8	108.2***
		Median	29	0.7	36	0.3	36	0.5	36	0.7	

(C) Pairwise comparison, χ^2

Sex	F	Strain/ Cross	Harwich F1	Harwich F70	Canton-S F1	Canton-S F70	♀H♂C-S F1	♀H♂C-S F70	♂C-S♀H F1	♂C-S♀H F70	Sex
	F1	Harwich	-	9.3*	44.4***	36.3***	234.5***	12.1**	213.4***	35.7***	Male
	F70		78.9***	-	78.3***	67.5***	284.5***	32.6***	240.8***	70.9***	
	F1	Canton-S	79.5***	140.1***	-	1.0	63.4***	7.9**	89.3***	1.3	
	F70		8.9**	63.0***	22.5***	-	59.3***	7.3**	77.4***	0.1	
	F1	♀H♂C-S	236.6***	141.8***	46.9***	0.0	-	114.7***	0.0	61.8***	
	F70		114.3***	75.7***	40.0***	8.9**	12.8***	-	98.1***	7.2**	
	F1	♂C-S♀H	152.4***	97.1***	34.1***	0.0	0.0	0.1	-	70.1***	
Female	F70		137.8***	49.3***	18.7***	0.0	0.0	0.1	0.0	-	

Notes: (A) Parent strains. Survival times were obtained for two strains kept at 29°C and fed by standard food either without or with adding different doses of caffeine-benzoate sodium; Averaged: See separate results for male and female flies in Figure S1; χ^2: Log Rank (Mantel-Cox) test of equality of survival distributions for two strains, df = 1, level of significance: *** $p < 0.001$. Log Rank (Mantel-Cox) test of equality of survival distributions for three doses: $\chi^2 = 25.8$ (x0 vs. x1), 89.3 (x0 vs. x10), and 14.2 (x1 vs. x10, $p < 0.001$ for all three pairwise comparisons of Harwich), and $\chi^2 = 0.5$ (x0 vs. x1, $p = 0.499$), 99.4 x0 vs. x10, $p < 0.001$, and 112.2 (x1 vs. x10, $p < 0.001$) for pairwise comparisons of Canton-S. Results are illustrated in Figures 2 and S2. (B) Parent strains and bidirectional crosses. Survival times were obtained for flies of two strains and their bidirectional crosses kept at 29°C; ♀H♂C-S: Crosses of females from Harwich strain with males from Canton-S strain; ♀C-S♂H: Crosses from female Canton-S strain with male Harwich strain (i.e., when females

are infertile under high developmental temperature). F (F1 and F70): Generation of intra-specific hybrids (1st and 70th); Me: Mean or Median. SEM: Standard Error of Me. Log Rank, χ^2: Four Log Rank (Mantel-Cox) tests of equality of survival distributions in strains/crosses, each sex in each of the experiments (with F1 and F70), df = 3. (C) Pairwise comparison, χ^2: Log Rank (Mantel-Cox) test for pairwise comparison of strains/crosses from the same sex in the same experiment with either F1 or F70, df = 15, level of significance: * $p < 0.05$, ** $p < 0.01$, *** $p < 0.001$.

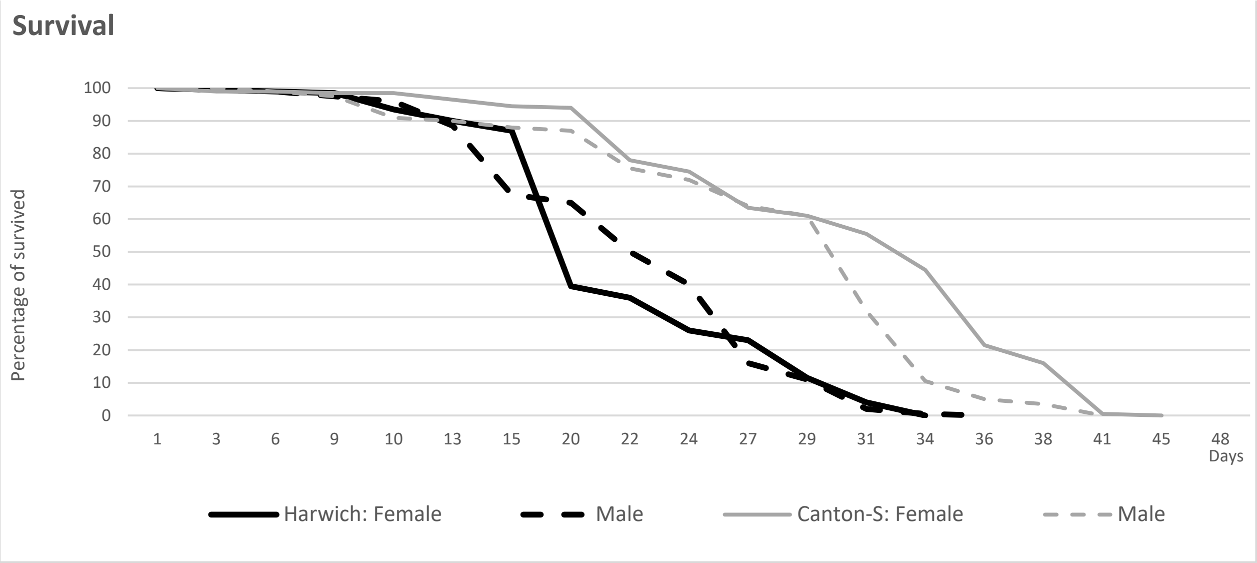

Figure S1. Survival curves obtained for male and female flies from two strains kept at 29°C. See statistical results in Table S1.

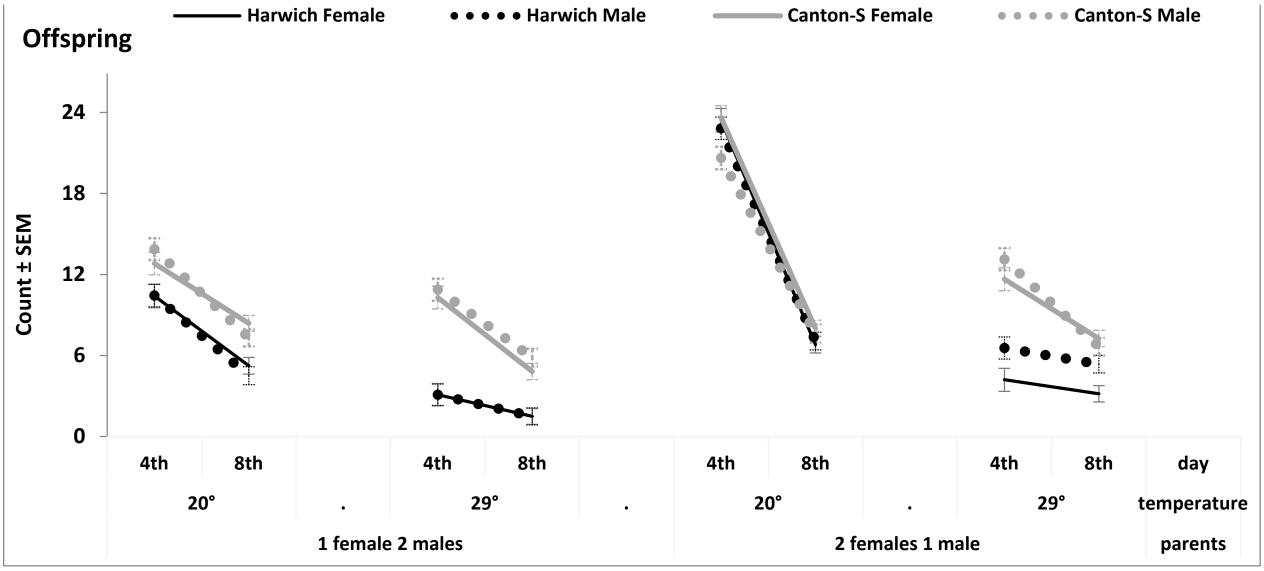

Figure S2. Fecundity under two different temperatures measured as the number of offspring per tube. Eggs laying lasted five days. Offspring were counted in 4th and 8th day of hatching imago. Parents were kept per tube in different proportions, either one male and two females or two females and one male from results of five-way rANOVA reported in Table S2 (left).

Table S2. Two rANOVAs of fecundity under different temperatures.

#	Factor	F	df	#	Factor	F	df
1	"Offspring's Sex"	0.6	1/240	1	"Offspring's Sex"	32.5***	1/104
2	"Day"	483.9***	1/240	2	"Day"	6.1*	1/104
3	"Parents' sex ratio"	170.7***	1/240	3	"Temperature"	75.1***	2/104
4	"Temperature"	368.2***	1/240	4	"Strain"	17.7***	1/104
5	"Strain"	119.7***	1/240		3*4	14.7***	2/104
	3*4	37.7***	1/240		1*2	10.6***	1/104
	3*5	9.7**	1/240		1*3	1.4	2/104
	4*5	38.7***	1/240		1*4	19.2***	1/104
	1*4	11.8***	1/240		2*3	0.9	2/104
	2*3	58.7***	1/240		2*4	55.6***	1/104
	2*4	126.9***	1/240		2*3*4	9.4***	2/104
	2*5	5.8***	1/240		1*2*3	18.9***	1/104
	1*2*5	7.3**	1/240		1*2*4	16.5***	2/104
	1*3*4	4.2*	1/240		1*3*4	12.1***	2/104
	2*3*4	62.7***	1/240		1*2*3*4	5.1**	1/104
	2*3	58.7***	1/240				
	2*4*5	15.7***	1/240				
	1*2*3*4	8.4***	1/240				

Notes: Left. Results of five-way rANOVA for fecundity under two different temperatures (non-significant results on interactions are omitted). Right. Results of four-way rANOVA for fecundity under three different temperatures. Fecundity was measured as the number of hatching imago per tube. The independent factors: "Parents' sex ratio" (one female and two males or two females and one male on the left), "Temperature" (20° or 29° on the left, and 20° or 25° or 29° on the right), and "Strain" (Harwich or Canton-S). Repeated measures: "Offspring's Sex" (male or female) and "Day" (two days of collection of offspring). F: F-ratio; df: Degrees of freedom were corrected using Greenhouse–Geisser correction controlling for type 1 errors associated with the violation of the sphericity assumption, but the original degrees of freedom are reported in this table. Level of significance: * $p < 0.05$, ** $p < 0.01$, and *** $p < 0.001$; #: Factor's order. Results are illustrated in Figures 3 (right) and S3 (left).

Table S3. ANOVAs and rANOVA of weight and two major insect sugars.

Two ANOVAs					rANOVA				
**Measure**	#	**Factor**	**F**	**df**	**Measure**	#	**Factor**	**F**	**df**
Fly weight, mg	1	"Sex"	224.3***	1/32	Sugars: glucose and trehalose, μg/mg fly	1	"Sugar"	2717.0***	1/32
	2	"Strain"	17.7***	1/32		2	"Sex"	15.6***	1/32
		1*2	0.0	1/32		3	"Strain"	83.6***	1/32
							2*3	18.9***	1/32
Trehalose–glucose ratio	1	"Sex"	88.1***	1/32			1*2	61.4***	1/32
	2	"Strain"	3.5*	1/32			1*3	18.9***	1/32
		1*2	0.0	1/32			1*2*3	3.5	1/32

Notes: Left. Results of two-way ANOVAs of fly weight and ratio between two main insect sugars. Right: Results of three-way rANOVA of concentrations of these two sugars. The independent factors: "Sex" (male or female) and "Strain" (Harwich or Canton-S). F: F-ratio; df: Degrees of freedom were corrected using Greenhouse–Geisser correction controlling for type 1 errors associated with the violation of the sphericity assumption, but the original degrees of freedom are reported in this table. Level of significance: * $p < 0.05$, and *** $p < 0.001$; #: Factor's order. Results are illustrated in Figures 3 and S3.

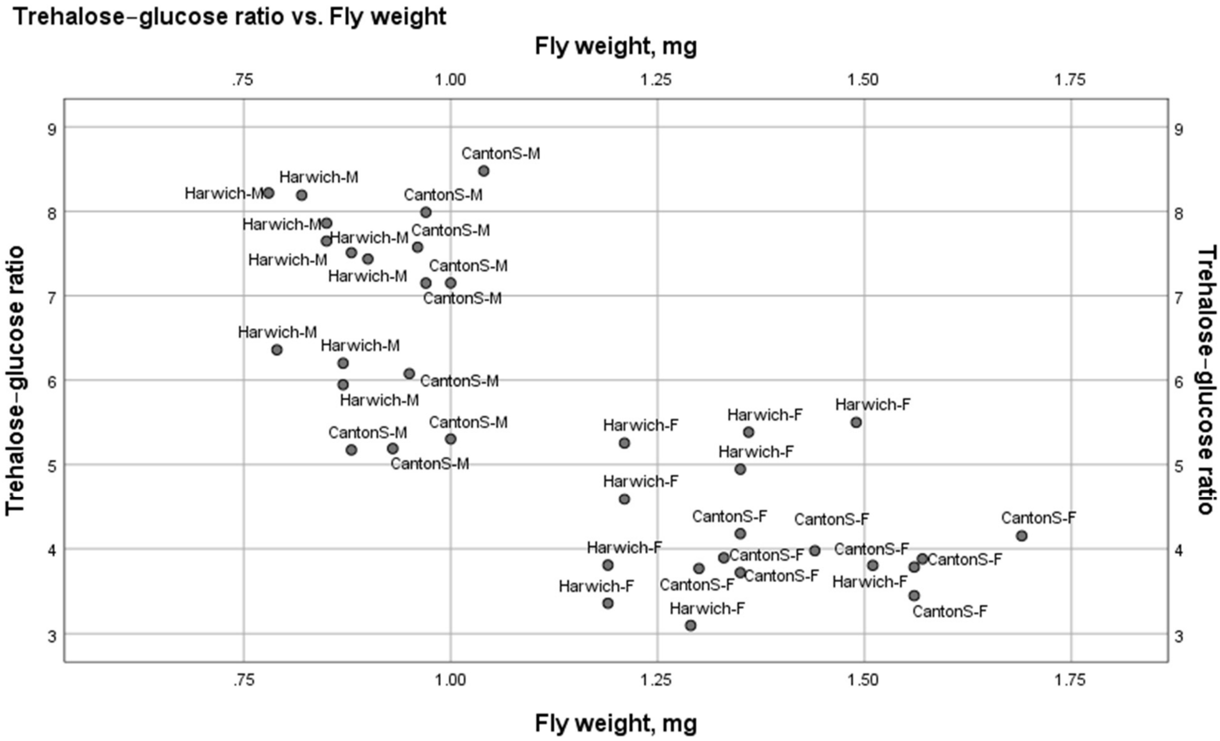

Figure S3. Body weight and ratio between two main insect sugars in male and female flies from two strains. Harwich-M and Harwich-F, and CantonS-M and CantonS-F: Harwich male and female, and Canton-S male and female. From the results of two two-way ANOVAs reported in Table S3 (left).

Table S4. Three- and four-way rANOVAs of locomotor activity and sleep.

#	Factor	F Activity	F Sleep	df	Factor	F Activity	F Sleep	df
Two three-way rANOVAs								
Strain (#2) +		+ age (#3)			Strain (#2) +	+ temperature (#3)		
1	"Time"	30.0***	57.3***	47/19,881	"Time"	19.3***	26.9***	47/2115
2	"Strain"	90.4***	88.8***	1/423	"Strain"	9.7***	10.2***	1/45
3	"Age"	7.0***	8.8***	3/423	"Temperature"	2.1	1.1	1/45
	2*3	3.2*	2.1	3/423	2*3	0.1	1.7	1/45
	1*2	7.2***	10.0***	1/19,881	1*2	6.1***	10.9***	47/2115
	1*3	5.1***	6.3***	141/19,881	1*3	4.5***	4.9***	47/2115
	1*2*3	2.0***	2.8***	141/19,881	1*2*3	2.5**	3.5***	47/2115
Four Four-way rANOVAs								
Strain (#2) +		+ age (#3) + dose (#4)			Strain (#2) +	+ temperature (#3) + dose (#4)		
1	"Time"	54.2***	107.7***	47/34,310	"Time"	22.2***	39.0***	47/8131
2	"Strain"	155.6***	217.0***	1/730	"Strain"	65.7***	134.8***	1/173
3	"Age"	35.8***	38.8***	1/730	"Temperature"	22.6***	16.5***	1/173
4	"Dose"	0.8	1.6	2/730	"Dose"	5.7*	4.4*	1/173
	2*3	0.0	0.9	1/730	2*3	17.2***	18.4***	1/173
	2*4	3.9*	8.8***	2/730	2*4	1.1	0.5	1/173
	3*4	0.7	2.2	2/730	3*4	2.0	0.3	1/173
	2*3*4	0.4	0.1	2/730	2*3*4	0.3	0.0	1/173
	1*2	20.3***	27.9***	47/34,310	1*2	8.1***	5.6***	47/8131
	1*3	25.0***	31.0***	47/34,310	1*3	15.5***	17.7***	47/8131
	1*4	4.3***	7.0***	94/34,310	1*4	3.3**	5.1***	47/8131
	1*2*3	2.9***	4.2***	47/34,310	1*2*3	8.5***	9.8***	47/8131
	1*2*4	3.6***	4.0***	94/34,310	1*2*4	0.7	0.9	47/8131
	1*3*4	1.4**	1.6***	94/34,310	1*3*4	1.1	1.6	47/8131
	1*2*3*4	1.5**	2.5***	94/34,310	1*2*3*4	0.8	0.6	47/8131
Strain (#2) +		+ food (#3) + dose (#4)			Strain (#2) +	+ cycle (#3) + dose (#4)		
1	"Time"	55.7***	101.1***	47/20,492	"Time"	117.7***	190.5***	47/13,771
2	"Strain"	102.6***	220.0***	1/436	"Strain"	133.9***	153.0***	1/293
3	"Food"	39.1***	91.9***	1/436	"Cycle"	0.6	6.5*	1/293
4	"Dose"	1.5	2.7	2/436	"Dose"	0.7	1.2	1/293
	2*3	16.8***	22.4***	1/436	2*3	0.1	0.9	1/293
	2*4	1.2	0.7	2/436	2*4	0.7	5.4*	1/293
	3*4	3.7*	7.4**	2/436	3*4	1.2	0.6	1/293
	2*3*4	1.3	0.7	2/436	2*3*4	1.1	0.4	1/293
	1*2	18.7***	21.2***	47/20,492	1*2	46.0***	57.6***	47/13,771
	1*3	9.4***	14.0***	47/20,492	1*3	20.2***	18.3***	47/13,771
	1*4	6.2***	11.4***	94/20,492	1*4	21.7***	30.0***	47/13,771
	1*2*3	6.6***	6.4***	47/20,492	1*2*3	6.6***	6.4***	47/13,771
	1*2*4	5.0***	6.9***	94/20,492	1*2*4	4.9***	3.6***	47/13,771
	1*3*4	1.5**	3.1***	94/20,492	1*3*4	1.9***	3.0***	47/13,771
	1*2*3*4	2.7***	4.0***	94/20,492	1*2*3*4	1.0	1.7	47/13,771

Notes: Six rANOVAs with the repeated measure "Time" (48 30-min intervals on the 24-h period). The independent factors: "Strain" (Harwich or Canton-S), "Age" (either four or two age categories), "Food" (either standard or low protein/high carbohydrate diet), "Temperature" (either 20° or 29° in all other cases), "Cycle" (either LD or DD in all other cases), "Dose" (food either without, x0, or with adding different doses of caffeine-benzoate sodium, either x2.5 or x10, or only x10); df: In rANOVA, degrees of freedom were corrected using Greenhouse–Geisser correction controlling for type 1 errors associated with the violation of the sphericity assumption, but the original degrees of freedom are reported in this table; F: F-ratio. Level of significance for F: * $p < 0.05$, ** $p < 0.01$, *** $p < 0.001$; #: Factor's order. Results are illustrated in Figures 4-9 and S4-S9.

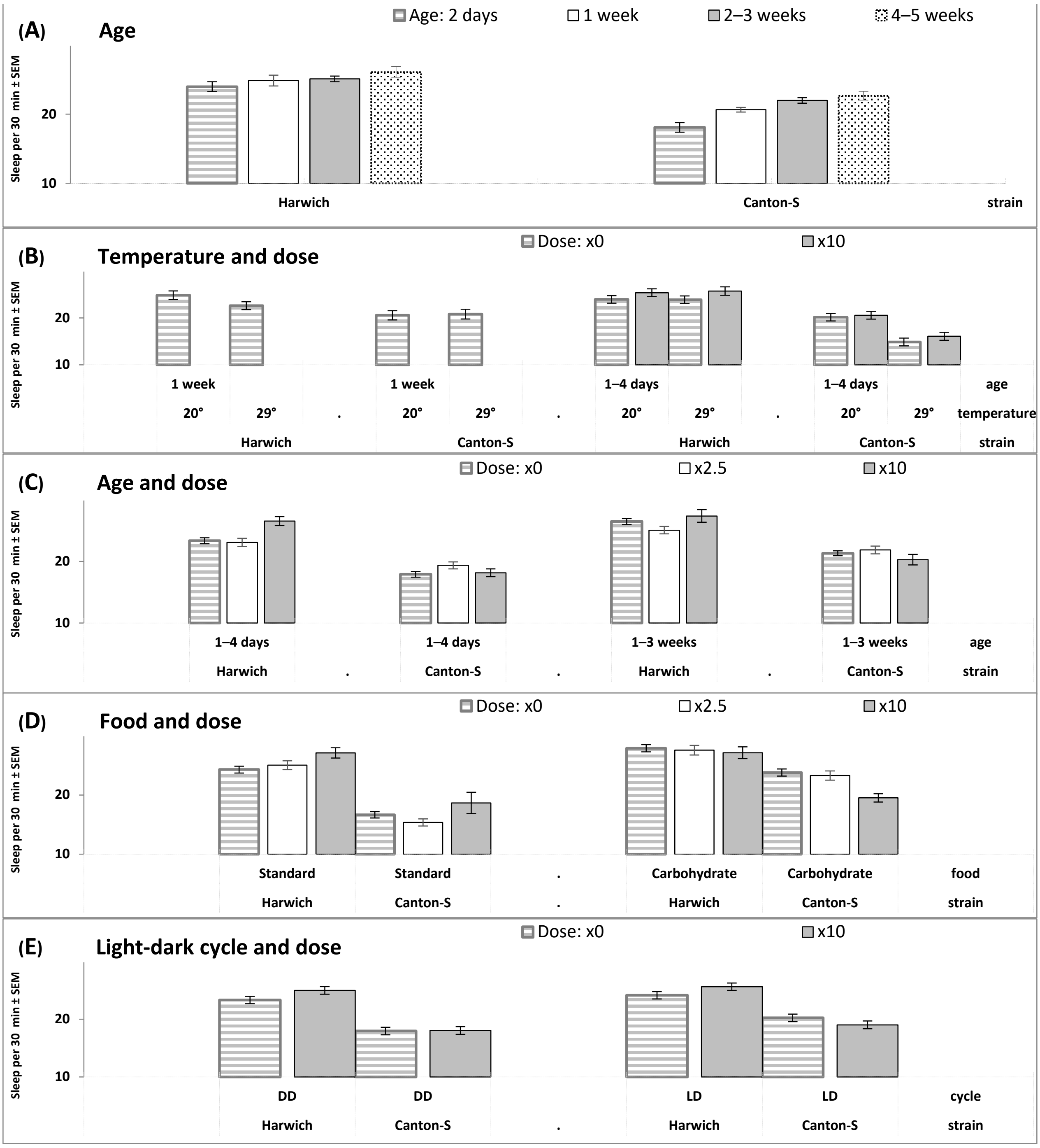

Figure S4. Effects on daily averaged sleep of aging, low and high temperature, carbohydrate and standard food, either without (x0) or with different doses of caffeine-benzoate sodium solution (x2.5 and x10), constant darkness (DD) and 24-h light-dark cycle (LD). See the 24-h patterns of sleep in Figures 5-9. This figure is based on the results of 6 rANOVAs reported in Table S4.

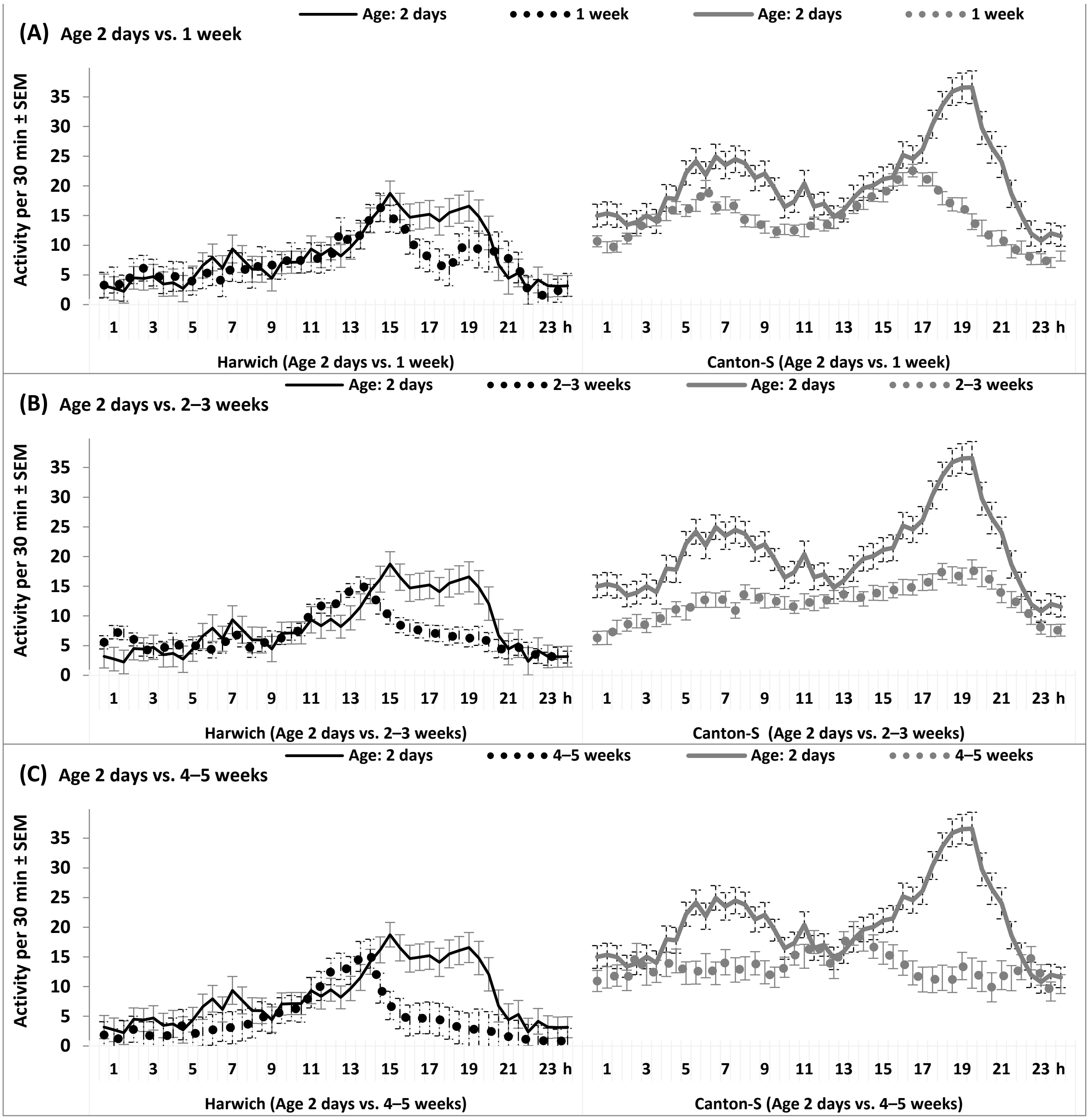

Figure S5. Effect of aging (from 2 days to 4-5 weeks) under high temperature on the 24-h pattern of locomotor activity. This and remaining figures are based on the results of one of rANOVAs reported in Table S4.

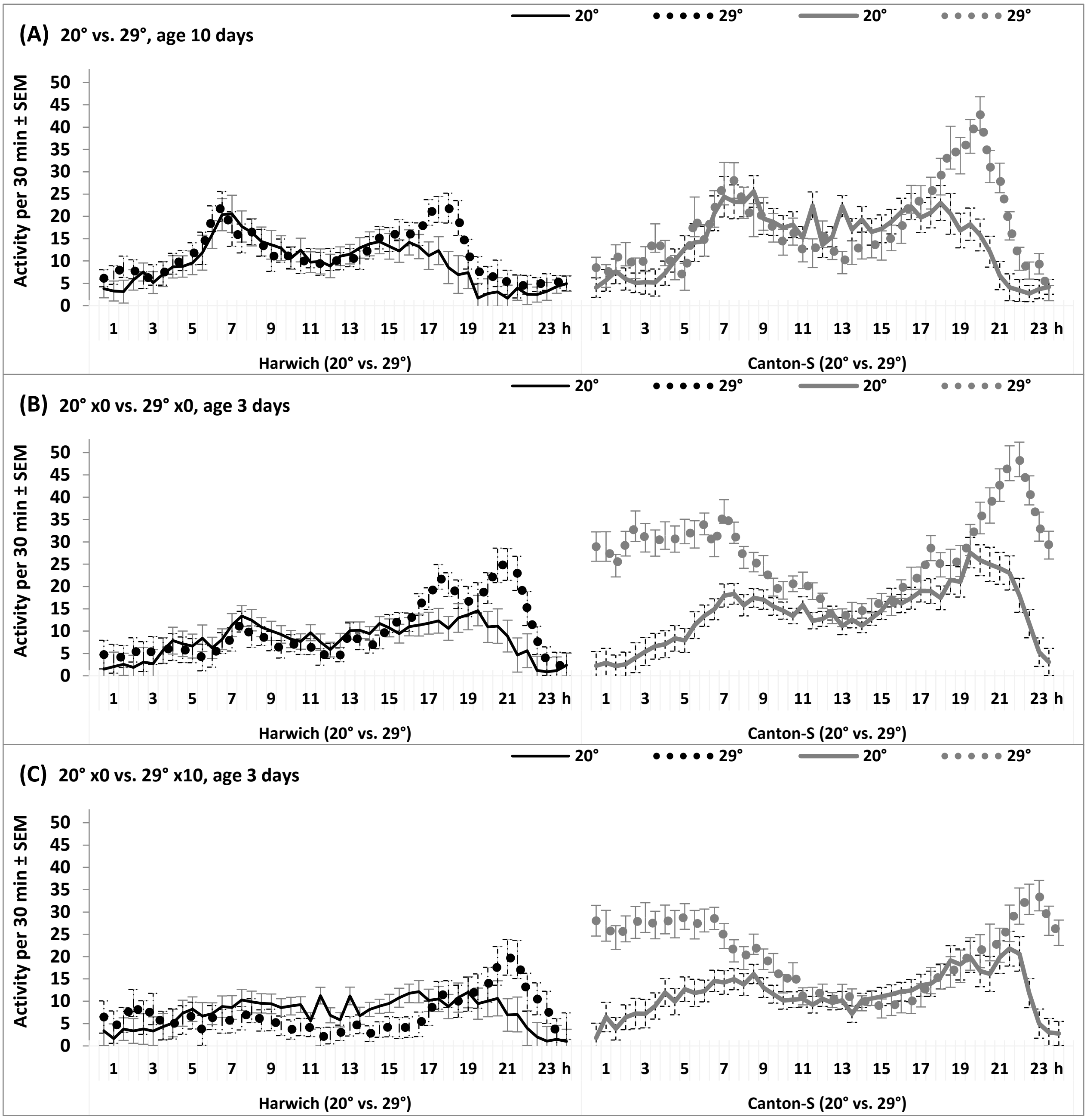

Figure S6. Effect low and high temperature (A), and effect of low and high temperature without or with 10 doses of caffeine-benzoate sodium on the 24-h pattern of locomotor activity. (A) Flies' age was 10 days. (B) and (C) Flies' age was 3 days. either standard food (dose x0) or standard food with adding 10 doses of caffeine-benzoate sodium, x10 (B and C, respectively).

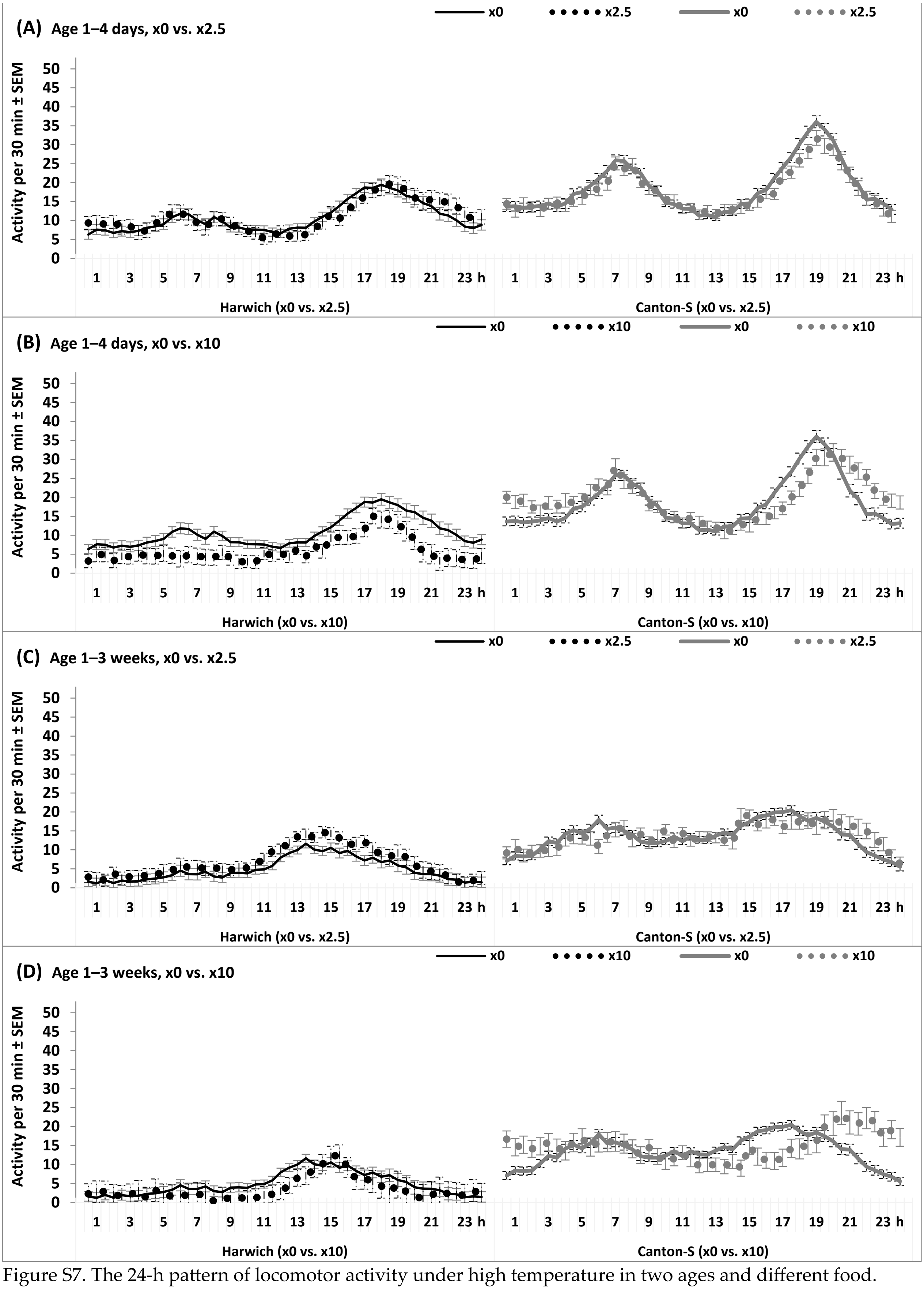

Figure S7. The 24-h pattern of locomotor activity under high temperature in two ages and different food.

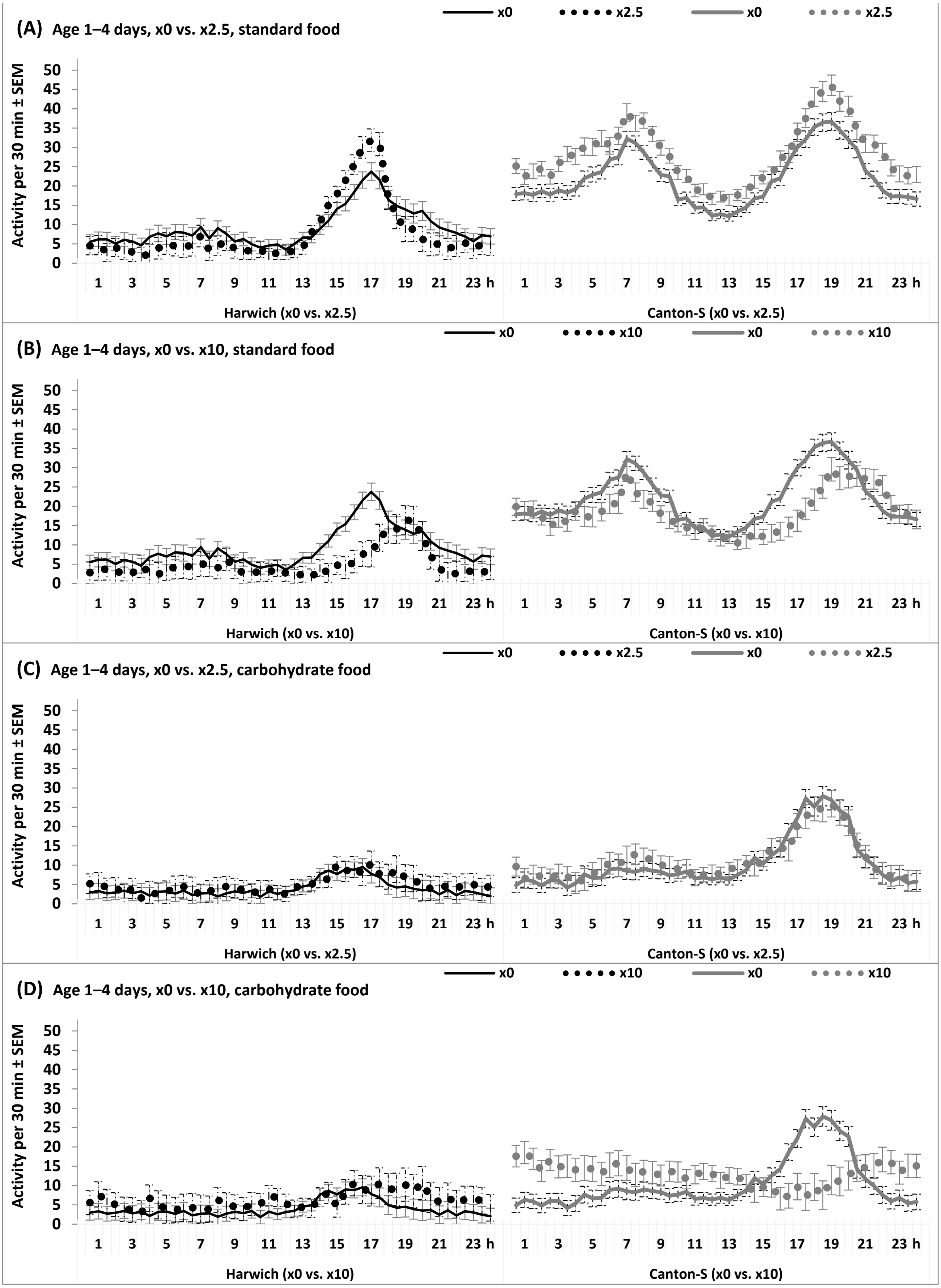
(A) Age 1–4 days, x0 vs. x2.5, standard food
x0
x2.5
x0
x2.5
Activity per 30 min ± SEM
0
5
10
15
20
25
30
35
40
45
50
1 3 5 7 9 11 13 15 17 19 21 23 h
1 3 5 7 9 11 13 15 17 19 21 23 h
Harwich (x0 vs. x2.5)
Canton-S (x0 vs. x2.5)
(B) Age 1–4 days, x0 vs. x10, standard food
x0
x10
x0
x10
Activity per 30 min ± SEM
Harwich (x0 vs. x10)
Canton-S (x0 vs. x10)
(C) Age 1–4 days, x0 vs. x2.5, carbohydrate food
x0
x2.5
x0
x2.5
Activity per 30 min ± SEM
Harwich (x0 vs. x2.5)
Canton-S (x0 vs. x2.5)
(D) Age 1–4 days, x0 vs. x10, carbohydrate food
x0
x10
x0
x10
Activity per 30 min ± SEM
Harwich (x0 vs. x10)
Canton-S (x0 vs. x10)

Figure S8. The 24-h pattern of locomotor activity under high temperature, either carbohydrate food or standard food without (x0) or with two doses caffeine-benzoate sodium solution (x2.5 and x10).

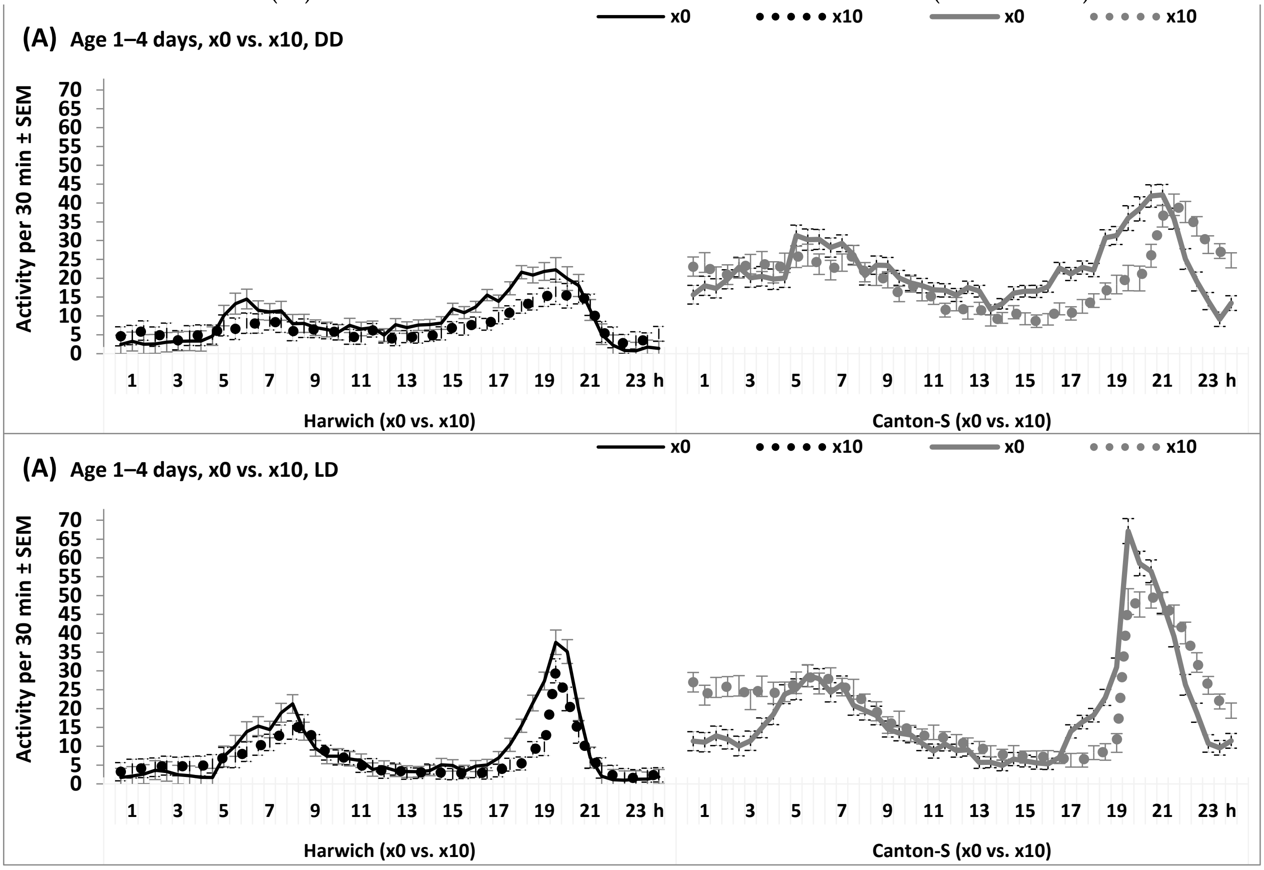

Figure S9. The 24-h pattern of locomotor activity in the condition of either constant darkness, DD, or under 24-h light-dark cycle, LD. Standard food without (x0) or with 10 doses of caffeine-benzoate sodium (x10).

Table S5. Rates of the 1st and 2nd principal component scores ≤ 0 and >0.

**PC score calculated**	**Age**	**Score either>0 or ≤0**			**Score either≤0 or >0**			
		**Harwich**	**Cross**	**Canton-S**	**Harwich**	**Cross**	**Canton-S**	χ^2
		PC1 or PC 2 score>0			PC1 or PC2 score≤0			
PC1 for activity	2 days	0.25	0.38	0.83	0.75	0.63	0.17	2.28
	1 week	0.11	0.20	0.75	0.89	0.80	0.25	19.80***
	2-3 weeks	0.19	0.25	0.57	0.81	0.75	0.44	28.18***
	4-5 weeks	0.09	0.24	0.60	0.91	0.76	0.40	18.39***
	Total	0.16	0.25	0.64	0.84	0.76	0.36	53.43***
PC2 for activity	2 days	0.39	0.50	0.21	0.61	0.50	0.79	3.56
	1 week	0.76	0.40	0.17	0.24	0.60	0.83	15.34***
	2-3 weeks	0.76	0.43	0.41	0.24	0.58	0.59	18.88***
	4-5 weeks	0.85	0.48	0.46	0.15	0.52	0.54	15.09**
	Total	0.72	0.45	0.37	0.29	0.56	0.63	41.19***
		PC1 or PC2 score>0			PC1 or PC2 score≤0			
PC1 for sleep	2 days	0.39	0.50	0.90	0.61	0.50	0.10	16.19***
	1 week	0.30	0.35	0.75	0.70	0.65	0.25	8.00*
	2-3 weeks	0.17	0.35	0.65	0.83	0.65	0.35	31.42***
	4-5 weeks	0.15	0.29	0.64	0.85	0.71	0.36	23.51***
	Total	0.23	0.34	0.70	0.77	0.66	0.30	76.01***
PC2 for sleep	2 days	0.43	0.38	0.24	0.57	0.63	0.76	19.85***
	1 week	0.76	0.35	0.08	0.24	0.65	0.92	20.43***
	2-3 weeks	0.70	0.20	0.33	0.31	0.80	0.67	22.50***
	4-5 weeks	0.88	0.45	0.46	0.12	0.55	0.54	26.56***

Total	0.70	0.34	0.34	0.30	0.66	0.66	87.68***

Notes: The 24-h patterns of locomotor activity and sleep were obtained for flies of two strains and their crosses (females of Canton-S strain were crossed with males of Harwich strainб ♀C-S♂H) under constant darkness at 29°C; scores on the 1st and 2nd principal components of variation in each of the 24-h patterns were calculated and dichotomized (either >0 or ≤0); χ^2: Chi-square test of equality of rates of dichotomized scores (either >0 or ≤0) for two strains and their crosses of each age, df = 2, level of significance: *** $p < 0.001$, ** $p < 0.01$, * $p < 0.05$. Similar differences are illustrated in Figure 10D (on the example of mean principal component scores calculated for each of strains/crosses of each of 4 ages).

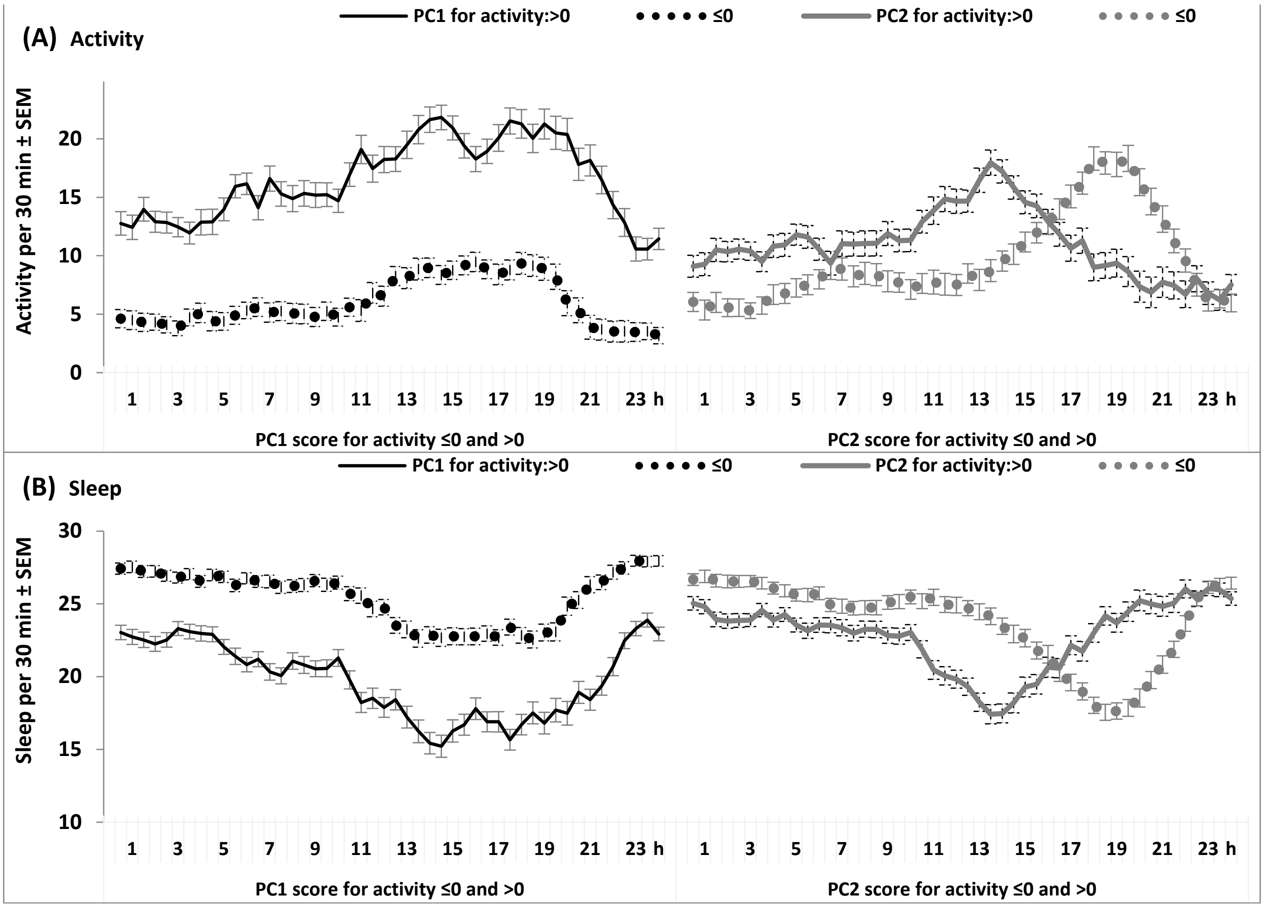

Figure S10. The 24-h patterns of activity and sleep for dihotomized PC1 and PC2 scores for activity.
(A) and (B) The 24-h patterns of activity and sleep, respectively, for dihotomized PC1 and PC2 scores for activity (either ≤0 or >0).

Supplementary Material 2: Details on Intraspecific Hybrid Dysgenesis.

Intraspecific PM hybrid dysgenesis (HD) of *Drosophila melanogaster* is characterized by gonadal atrophy in one of the cross-directions as a result of nuclear-cytoplasmic incapability [1]. When all other things being equal, the ability to induce HD is the prerogative of a limited number of reference strains. Mainly, Harwich is used as inducer strain and Canton-S is used as reactive strain. According to our data, the strains differ in key physiological parameters. For all indicators of vitality, Harwich is an inferior strain compared to Canton-S. PM HD gonadal atrophy is considered to be caused by the massive movement of the P-elements that were introduced in the *D. melanogaster* genome in the middle of the last century [2].

However, we found that there are published results conflicting with the concept postulating that P-element transpositions are responsible for PM HD in *D. melanogaster*. The arguments might be the following: 1. Lack of asymmetry in P-element content in most parental lines [3-4]. 2. Low speed of TEs movement [5-6]. 3. Weak influence of temperature on TEs activity [7]. 4. Rapid expansion of P-element in natural populations of *D. melanogaster* and *D. simulans* [8-9]. 5. Uniqueness of Harwich as the reference for PM HD-inducing strain [3-4]. The presence of P-element in the genome is more likely a marker of genetic distance between parental strains rather than a cause of HD. Further research is needed to elucidate the causes of HD and their contribution to gonadal atrophy.

References

1. Kidwell, M.G.; Kidwell, J.F. Cytoplasm-chromosome interactions in *Drosophila melanogaster*. *Nature* **1975**, *253*, 755–756. http://doi.org/10.1038/253755a0
2. Kidwell, M.G. Hybrid dysgenesis in *Drosophila melanogaster*: nature and inheritance of P element regulation. *Genetics* **1985**, *111*, 337-350. http://doi.org/10.1093/genetics/111.2.337.
3. Itoh, M.; Fukui, T.; Kitamura, M.; Uenoyama, T.; Watada, M.; Yamaguchi, M. Phenotypic stability of the P-M system in wild populations of *Drosophila melanogaster*. *Genes Genet. Syst.* **2004**, *79*, 9-18. http://doi.org/10.1266/ggs.79.9.
4. Ignatenko, O.M.; Zakharenko, L.P.; Dorogova, N.V.; Fedorova S.A. P elements and the determinants of hybrid dysgenesis have different dynamics of propagation in *Drosophila melanogaster* populations. *Genetica* **2015**, *143*, 751-759. http://doi.org/10.1007/s10709-015-9872-z.
5. Keightley, P.D.; Ness, R.W.; Halligan, D.L.; Haddrill, P.R. Estimation of the spontaneous mutation rate per nucleotide site in a *Drosophila melanogaster* full-sib family. *Genetics.* **2014**; *196*, 313–320. http://doi.org/10.1534/genetics.113.158758
6. Mérel, V.; Boulesteix, M.; Fablet, M.; Vieira, C. Transposable elements in *Drosophila*. *Mob. DNA* **2020**; *11*, 23. http://doi.org/ 10.1186/s13100-020-00213-z.
7. Mombach, D.M.; da Fontoura Gomes, T.M.F.; Loreto, E.L.S. Stress does not induce a general transcription of transposable elements in *Drosophila*. *Mol. Biol. Rep*. **2022**, *49*, 9033–9040. https://http://doi.org/10.1007/s11033-022-07839-7.
8. Kidwell, M.G. Hybrid dysgenesis in *Drosophila melanogaster*: factors affecting chromosomal contamination in the P-M system. *Genetics* **1983**, *104*, 317-341. http://doi.org/10.1093/genetics/104.2.317.
9. Hill, T.; Schlötterer, C.; Betancourt, A.J. Hybrid dysgenesis in *Drosophila simulans* associated with a rapid invasion of the P-element. *PLoS Genet*. **2016**, *12*, e1005920. http://doi.org/10.1371/journal.pgen.1005920.
